# Perspectives of RNAi, CUADb and CRISPR/Cas as Innovative Antisense Technologies for Insect Pest Control: From Discovery to Practice

**DOI:** 10.3390/insects16070746

**Published:** 2025-07-21

**Authors:** Hemant Kumar, Nikita Gal’chinsky, Verma Sweta, Nikita Negi, Roman Filatov, Anamika Chandel, Jamin Ali, Vol Oberemok, Kate Laikova

**Affiliations:** 1 Department of Entomology, Agriculture College, Garhwa, Birsa Agricultural University, Ranchi 834006, India; hksahu.iari@gmail.com; 2 Department of General Biology and Genetics, V.I. Vernadsky Crimean Federal University, 295007 Simferopol, Russia; variant_022@mail.ru (R.F.); voloberemok@gmail.com (V.O.); botan_icus@mail.ru (K.L.); 3 Division of Entomology, ICAR-Indian Agricultural Research Institute, New Delhi 110012, India; drsvbiotech@gmail.com; 4 Division of Genomic Resources, National Bureau of Agricultural Insect Resources, Bengaluru 560024, India; nikinegi10@gmail.com; 5 Division of Vegetable Sciences, ICAR-Indian Agricultural Research Institute, New Delhi 110012, India; anamikathakuriari@gmail.com; 6 College of Plant Protection, Jilin Agricultural University, Changchun 130118, China; j.alirana@yahoo.com

**Keywords:** RNAi, CUADb, CRISPR/Cas, antisense technologies, insect pest control

## Abstract

Pest management is undergoing a transformative shift with the development of the cutting-edge antisense technologies: RNA interference (RNAi), contact unmodified antisense DNA biotechnology (CUADb), and the CRISPR-associated proteins (CRISPR/Cas). These approaches function by facilitating sequence-specific pairing of nucleic acids followed by nuclease-mediated cleavage, offering exceptional precision for targeted pest control. While RNA-guided mechanisms such as RNAi and CRISPR/Cas were initially characterized in non-insect systems, primarily as innate defenses against viral infections, the DNA-guided CUADb pathway was first identified in insect pests as a functional pest control strategy. Its broader role in ribosomal RNA (rRNA) biogenesis was recognized later. Together, these discoveries have revealed an entirely new dimension of gene regulation, with profound implications for sustainable pest management. Despite sharing a common principle of sequence-specific targeting RNAi, CUADb, and CRISPR/Cas differ in several key aspects, including their mechanisms of action, target specificity, and applicability. Rather than serving as universal solutions, each technology is likely to be optimally effective against specific pest groups. Moreover, these technologies allow for rapid adaptation of control strategies to overcome target-site resistance, ensuring long-term efficacy. This review summarizes the core functional characteristics, potential applications, and current limitations of each antisense technology, emphasizing their complementary roles in advancing environmentally sustainable pest control. By integrating foundational biological discoveries with applied innovations, this work provides a new perspectives on incorporating antisense-based strategies into next-generation integrated pest management systems.

## Simple Summary

RNA interference (RNAi), contact unmodified antisense DNA biotechnology (CUADb), and clustered regularly interspaced short palindromic repeats (CRISPR) along with CRISPR-associated proteins (CRISPR/Cas) represent three innovative cost-effective antisense technologies for insect pest control. Each method operates through the formation of specific unmodified nucleic acid complexes, RNAi involves guide RNA binding to messenger RNA (mRNA); CUADb: employs guide DNA interacting with ribosomal RNA (rRNA); and CRISPR/Cas: utilizes guide RNA targeting genomic DNA. These complexes function via nucleic acid-guided nucleases: Argonaute in RNAi; rRNase in CUADb, and CRISPR-associated proteins in CRISPR/Cas systems.

## 1. Introduction

Nucleic acids, DNA and RNA, orchestrate cellular processes through precise complementary interactions [1]. The fundamental principles of Watson–Crick base pairing, combined with the action of specific enzymes, govern essential biological mechanisms such as replication, transcription, translation, and regulation of gene expression [2]. The specificity and fidelity of these processes arise from the unique combinations of nitrogenous bases, which constitute the molecular foundation of genetic control. Three innovative antisense technologies, RNA interference (RNAi) [3], contact unmodified antisense DNA biotechnology (CUADb) [4,5,6], and clustered regularly interspaced short palindromic repeats (CRISPR) along with CRISPR-associated proteins (CRISPR/Cas) [7,8,9], have harnessed these nucleic acid interactions to induce targeted genetic effects. These technologies operate through the formation of sequence-specific duplexes: RNAi (guide RNA–messenger RNA (mRNA)) [10], CUADb (guide DNA-ribosomal RNA (rRNA)) [5,11,12,13,14], and CRISPR/Cas (guide RNA-genomic DNA) [9,15,16], which subsequently recruit specialized nucleases such as Argonaute (Ago) [17], rRNase [5,6], and CRISPR-associated proteins [9].

**Figure 1:**
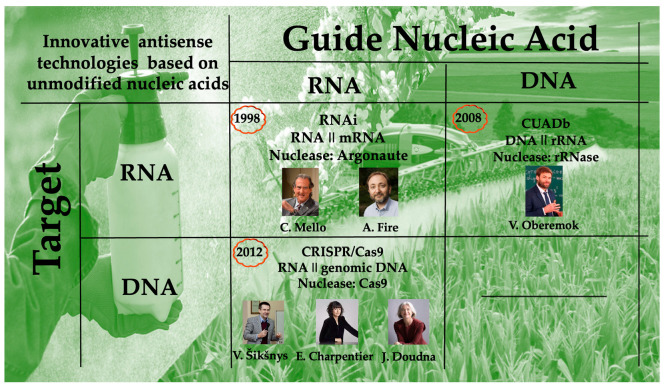
Antisense technologies based on unmodified nucleic acids and used for insect pest control: guide nucleic acid (RNA or DNA) forms duplex with target nucleic acid (RNA or DNA) and the target nucleic acid is cleaved by specific nuclease.

The emergence of these technologies was made possible by significant advances in bioinformatics, in vitro nucleic acid synthesis, and the development of modern molecular tools. Typically, a pivotal insight or an experimental breakthrough reveals a pattern that can be reliably applied in practice. For RNAi, the key finding was the use of an antisense fragment within double-stranded RNA (dsRNA); for CUADb, it was the understanding that antisense oligodeoxyribonucleotides can be used as contact insecticides; and for CRISPR/Cas, it was the identification that genomic DNA could serve as the target for antisense-guided effects. These innovations have set new standards in molecular genetics and are now being widely explored for their applications in insect pest control. Each of these technologies emerged from fundamental research, evolving from uncertain beginnings into powerful tools with transformative potential. Since their core mechanism relies on the complementary binding of antisense molecule, either DNA or RNA, to a target nucleic acid, they are collectively referred to as antisense technologies (Figure 1). While RNA- and DNA-guided nucleases have been extensively studied, a targeted DNA-cleaving mechanism utilizing guide DNA to cut pest genomic DNA via a specific nuclease has not yet been developed, representing a potential direction for future research.

Despite the role of chemical insecticides is pest management [18], several factors drive the search for alternative control strategies. The most important of these is high economic cost of insect pest damage to agriculture and growing prevalence of insecticide resistance, which has increased dramatically and persistently since the mid-20th century [18,19,20]. Insecticide resistance generally arises through natural selection, whereby the frequency of specific alleles increases due to random mutations within pest populations [21]. Antisense technologies (RNAi, CUADb, and CRISPR/Cas) are able to counteract insecticide resistance by targeting conserved genes or conserved gene regions and by facilitating the rapid development of effective pest control agents when resistance to existing insecticides emerges. While CUADb [6] and RNAi [3] demonstrate promising potential as next-generation bioinsecticides, due to their fast biodegradability, selectivity, and low carbon footprint [12], CRISPR/Cas is primarily used to genetically attenuate insect pest populations through genetic engineering [7]. Nevertheless, these innovative antisense approaches, and their potential combinations, offer a broad and flexible toolkit for insect pest control. The primary challenge lies in selecting the most suitable strategy for each specific pest management scenario.

The development of antisense technologies was predicted on the discovery of the DNA double helix, followed by a formative period marked by pioneering studies, such as the site-specific modification of valine tRNA by Grineva and colleagues [22] and the work of Zamecnik and Stephenson using modified DNA against the *Rous sarcoma* virus [23]. However, it became critically important to develop methods employing unmodified (natural) nucleic acids and to transform them into cost-effective, environmentally safe antisense technologies. While the RNAi was discovered by a research group in the USA in 1998 [3], CUADb was introduced in 2008 and subsequently developed further in Crimea [4,6], the development of the CRISPR/Cas9 was established in 2012 through the contributions of several research groups, primarily from Lithuania, Sweden and the United States [7,24,25]. These efforts collectively lead to the establishment of the three main antisense technologies for pest control at the turn of the 21st century. Although CUADb offers a relatively straightforward framework for pesticide design, RNAi and CRISPR/Cas are still progressing toward streamlined, reliable strategies for creating selective and effective pest control agents. The objective of this review is to provide a concise historical overview of the emergence of antisense technologies and to assess their potential in insect pest control. Overall, we provide an evaluation of the current capabilities and limitations of antisense technologies in this field, and propose directions for future development.

## 2. RNAi

### 2.1. History of Discovery of RNAi

**Figure 2:**
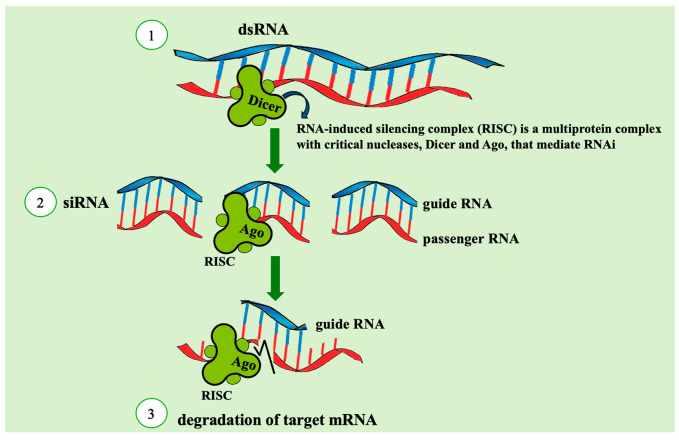
Main route of RNAi used for creation of dsRNA insecticides (exogenous siRNA pathway): 1–dsRNA cleavage by Dicer; 2–silencing complex formation and silencing complex activation; 3–target mRNA degradation by Ago.

The discovery of RNAi was inspired by the pioneering work of Zamecnik and Stephenson (1978), who demonstrated that a short antisense sequence of modified nucleic acid could inhibit the replication of the *Rous sarcoma* virus [23]. In 1998, Mello and Fire investigated the effect of both antisense and sense RNA fragments on the development of the nematode *Caenorhabditis elegans*. Their aim was to elucidate the mechanisms underlying the efficacy of sense RNA fragments synthesized by bacteriophage RNA polymerase (specifically, DNA-dependent RNA polymerases), using dsRNA fragments as experimental controls. Bacteriophage polymerases, although highly specific, occasionally produce ectopic transcripts, and DNA transgene arrays, also known as extrachromosomal arrays, are known to generate aberrant RNA products. Mello and Fire hypothesized that the interfering RNA population might include molecules with double-stranded features. Unexpectedly, it was the dsRNA fragment that triggered a potent silencing effect on the endogenous *mex-3* mRNA transcript, which is highly expressed in the gonads and early embryos of the nematode. The groundbreaking discovery, published in *Nature* in 1998 [3], established the foundational concept of RNAi: dsRNA molecules can initiate gene silencing by targeting homologous transcripts [26,27]. Subsequent research revealed that short antisense RNA fragments (21–23 nucleotides in length) are produced during RNAi and mediate the degradation of target mRNAs [28] (Figure 2).

### 2.2. How RNAi Works in Insect Pests

RNAi has been extensively studied for its potential in agricultural pest management, particularly through the silencing of essential insect genes to suppress pest populations. The small interfering RNA (siRNA) pathway, the most thoroughly characterized RNAi mechanism, consists of two branches: the exogenous siRNA (exo-siRNA) pathway, which mediates antiviral defense, and the endogenous siRNA (endo-siRNA) pathway, responsible for transposon suppression within the genome [29]. The exo-siRNA pathway is commonly utilized in experimental and applied contexts by introducing dsRNA to silence specific target genes [30,31]. For effective gene silencing, long dsRNA fragments (>200 bp) are typically used to trigger the production of short antisense siRNAs [26,27]. The RNAi process begins with cellular uptake of dsRNA, followed by cleavage into siRNAs (21–25 nucleotides) by the enzyme Dicer. These siRNAs are then incorporated into the RNA-induced silencing complex (RISC), where Argonaute (Ago) proteins serve as the catalytic core [32]. One strand of the siRNA duplex, known as the guide strand, directs RISC to the complementary mRNA target, leading to transcript degradation or translational repression [33].

Although much of the research has focused on the siRNA machinery, the RNAi pathway in insects is more complex and includes three sub-pathways: siRNA, micro (miRNA), and piwi-interacting RNA (piRNAs). miRNAs (18–24 nucleotides) are involved in diverse regulatory processes and interact with Ago proteins, while piRNAs (26–31 nucleotides) are primarily responsible for silencing transposable elements, particularly in germline cells, through interactions with PIWI proteins. Artificially synthesized dsRNA may influence not only the siRNA pathway but also genes associated with the miRNA pathway, suggesting a broader, interconnected regulatory network [34].

RNAi is particularly robust and systemic in Coleopteran insects [29]. However, systemic RNAi, defined as the environmental uptake of dsRNA followed by intercellular transport, is limited in Diptera, Lepidoptera [35,36,37], and sap-feeding Hemiptera [38,39], thereby constraining its utility in some agricultural contexts. Nonetheless, RNAi has demonstrated considerable potential for control of holometabolous and hemimetabolous insect pests, by targeting key genes involved in development, detoxification, and reproduction (Table 1).

### 2.3. Perspectives and Limitations of RNAi for Insect Pest Control

Despite significant advancements in RNAi research over the past 25 years, its practical management of insect pests and disease vectors remains limited. To date, only one commercial dsRNA-based biopesticide, Calantha^®^, has been developed for foliar application, with several others currently under development. Additionally, three transgenic crops expressing dsRNA are commercially available.

Calantha^®^, a sprayable dsRNA-based biopesticide targeting the Colorado potato beetle (CPB, *Leptinotarsa decemlineata*), was released commercially by GreenLight Biosciences in 2023 [68,69]. As with dsRNA-expressing crops, sprayable formulations are likely to be used in combination with other pest control tools. However, sprayable dsRNAs degrade rapidly in the environment [70], necessitating careful consideration of application timing, spray intervals and synchronization with insect life cycle to ensure that only a single generation is exposed to the dsRNA product. Subsequent insecticide treatments should then be rotated to a different mode of action [71,72].

Several challenges have hindered the widespread commercialization of RNAi-based products. These include variable efficacy across insect species, competition from transgenic Bt crops, and limited effectiveness against some sap-sucking pests. Another concern is the potential evolution of RNAi resistance. For example, studies have shown that western corn rootworm (*Diabrotica virgifera virgifera*) [73], willow leaf beetle (*Plagiodera versicolora*) [74], and CPB [75,76] can develop resistance to dsRNA-based control. Of note, Calantha^®^ can be deployed in pesticide rotation programmes to mitigate selection for resistance in CPB [77]. Nonetheless, RNAi, when combined with precision agriculture and integrated pest management (IPM) strategies, could significantly enhance sustainability. Opportunities for synergy exist with advancing the technologies such as CUADb, CRISPR/Cas technology, microbial-based RNAi production, and nanoformulations for improved dsRNA delivery. Of note, RNA molecules are prone to degradation in the environment. To enhance their effectiveness, researchers are developing more stable RNA formulations and innovative delivery methods.

A clear understanding of dsRNA structure is critical for optimizing RNAi applications in pest control. This structural knowledge informs how dsRNA is processed into siRNAs and facilitates the precise targeting of pest genes for silencing [78,79]. Although the effects of dsRNA-mediated interference are highly potent and specific, there are several concerns that should be taken into account when designing RNAi-based experiments. If a sequence is shared among multiple closely related genes, RNAi may unintentionally silence several members of the gene family or homologous genes in related species [80]. Also, genes with low expression levels may exhibit resistance to RNAi, at least partially. For example, if the target protein is very stable, its depletion occurs much more slowly despite transcript degradation. Moreover, the function of a target protein can be compensated for by related proteins, which may even be upregulated by the cell to counteract the loss. Additionally, the transcription of the target gene itself could be upregulated by a regulatory gene network in response to RNAi mediated knock-down [81].

Importantly, insights into how Argonaute proteins bind to guide and target RNAs allow for the rational design of highly specific and effective RNAi tools. Structural analyses of the Argonaute active site and its interaction with target RNAs enable the optimization of siRNA sequences for enhanced gene silencing [82,83]. Furthermore, understanding the architecture of RISC components supports improvements in delivery strategies and silencing efficiency [84]. Overall, this structural knowledge is essential for developing RNAi-based pest control methods that are both species-specific and environmentally sustainable [85].

Currently, the implementation of RNAi-based pest control strategies is challenged by regulatory uncertainties and public apprehension. Gaining regulatory approval and public acceptance will depend on transparent scientific communication, rigorous safety evaluations, and evidence-based demonstrations of efficacy. A major practical limitation remains the high cost of dsRNA production; publicly accessible in vitro synthesis methods are still prohibitively expensive, exceeding $50 per milligram [86,87,88,89,90].

Despite these challenges, RNAi remains a promising strategy for insect pest control due to its selectively silence genes critical for insect development and survival. This specificity provides an opportunity to develop environmentally sustainable pest control solutions. However, the successful deployment of dsRNA-based insecticides will require coordinated efforts from researchers, policymakers, and industry stakeholders worldwide. Future advancements, particularly in dsRNA formulation, delivery systems, and cost-effective production, are expected to drive the broader adoption of RNAi in the coming decades, potentially transforming agricultural pest management practices [91].

## 3. CUADb

### 3.1. History of Discovery of CUADb

Unmodified DNA, as a programmable molecule and natural biopolymer, has long captured scientific interest. However, for many years it was believed that unmodified oligodeoxyribonucleotides were both cytotoxic and highly susceptible to degradation in eukaryotic cells due to nuclease activity [92], including in insects [11,93]. Some studies explicitly stated that unmodified (phosphodiester) antisense oligonucleotides should not be employed in experimental applications [94]. Additionally, it was widely assumed that ribosomal RNA (rRNA) was inherently resistant to degradation by antisense DNA oligonucleotides [95,96]. At the turn of the century, there was no indication in the scientific literature that DNA could be used as an insecticide.

**Figure 3:**
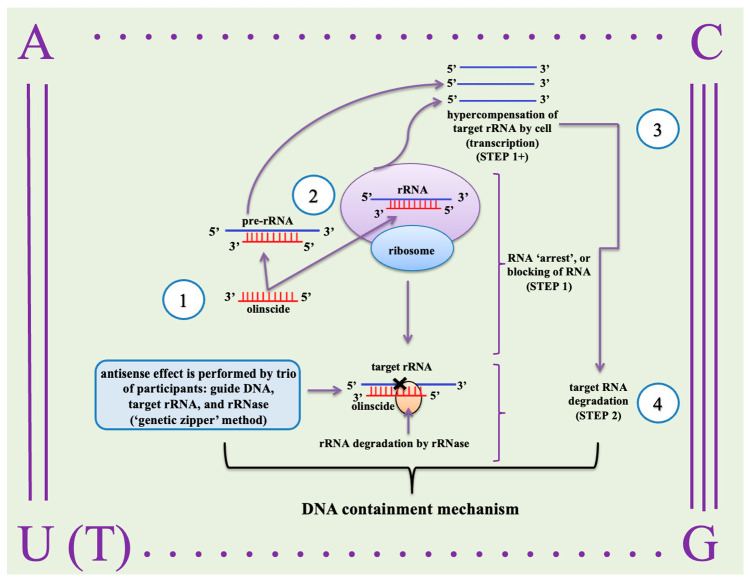
DNA containment (DNAc) is the main mechanism of action of oligonucleotide insecticides (olinscides): 1—antisense DNA sequences complementary to rRNA and/or pre-rRNA of pests are used as oligonucleotide insecticides; 2—formation of duplex between DNA (oligonucleotide insecticide) and RNA (rRNA and/or pre-rRNA) of the pests; 3—at first step of DNAc, antisense DNA oligonucleotide (oligonucleotide insecticide) ‘arrests’ target rRNA and/or pre-rRNA followed by its hypercompensation and interferes with normal functioning and biogenesis of ribosomes (‘arrested’ ribosomes); 4—at second step of DNAc, rRNase cleaves target rRNA and/or pre-rRNA and substantial decrease in its concentration occurs.

The insecticidal properties of DNA were discovered serendipitously in the spongy moth (*Lymantria dispar*). In 2007, Oberemok initiated research on the transovarial transmission of *L. dispar* multiple nucleopolyhedrovirus (LdMNPV) as part of his doctoral work. Two specific primers were selected within the anti-apoptotic gene (IAP-3) of LdMNPV: a forward primer from the sense strand (5′-GCCGGCGGAACTGGCCCA-3; oligoBIR fragment) and a reverse primer from the antisense strand (5′-CGACGTGGTGGCACGGCG-3′; oligoRING fragment) [97]. These primers generated a 317 bp amplicon in PCR using purified viral DNA [98]. However, when applied to DNA extracted from virus-free *L. dispar*, the primers produced amplicons of varying lengths, suggesting non-specificity and indicating the presence of homologous sequences in the host genome—a phenomenon previously documented for other viruses [99]. Given this, it was hypothesized that the oligoBIR and oligoRING fragments could modulate the expression of homologous *L. dispar* IAP genes, potentially inducing apoptosis. In April 2008, Oberemok tested this hypothesis unconventionally by topically applying drops of aqueous solutions of the primers to the surface of *L. dispar* larvae [4,100]. Remarkably, after 3–5 days, significant larval mortality was observed, attributable to the applied DNA fragments derived from the viral genome. This experiment marked the inception of a new class of contact DNA insecticides, termed oligonucleotide insecticides, or briefly olinscides, and the CUADb (Contact Unmodified Antisense DNA biotechnology) platform. The first results were reported in a Ukrainian patent (No. 36445) in 2008, followed by articles in Pesticide Biochemistry and Physiology [101,102]. The earliest oligonucleotide insecticides (18–20 nt) targeting anti-apoptotic genes were effective in both virus-free and LdMNPV-infected larvae [97]. Importantly, unique antisense DNA sequences of 11–20 nt offer high target selectivity, although their efficacy depends on the abundance of target RNA. Consequently, CUADb was optimized to target rRNA—which constitutes approximately 80% of total cellular RNA—via a DNA containment (DNAc) mechanism, resulting in a robust algorithm for insect pest control (Figure 3). This concept later was used by RNAi researchers. Notably, three years after Oberemok’s discovery, Wang et al. [103] successfully demonstrated, for the first time, the use of dsRNA fragments as contact insecticides in pest control.

### 3.2. How CUADb Works on Insect Pests

In 2019, Oberemok and colleagues introduced several major refinements to the CUADb platform aimed at enhancing its efficacy and cost-effectiveness. A key advancement was the selection of insect ribosomal RNA (rRNA) as the primary target for oligonucleotide insecticides [11]. Targeting pre-rRNA and mature rRNA significantly enhances efficacy because these RNA molecules account for approximately 80% of total cellular RNA [104], in contrast, mRNAs, which constitute only around 5%. This strategic targeting substantially increases the signal-to-noise ratio, up to approximately 100,000:1 when compared to random mRNA sequences. Insects possess both nuclear and mitochondrial rRNA, including 28S (~3900 nucleotides (nt)), 18S (~1920 nt), 5.8S (~160 nt), and 5S (~120 nt), 16S (~1140 nt) and 12S (~600 nt) making rRNA an ideal and abundant molecular target.

A second major refinement involved reducing the length of oligonucleotide insecticides to 10–12 nt to lower production costs. Shorter sequences result in higher yields during phosphoramidite-based DNA synthesis increasing product mass per synthesis cycle. Despite their brevity, 11-nt oligonucleotides retain sufficient specificity, with a uniqueness frequency of approximately 1 in 4.19 million sequences, adequate for most agricultural systems [105]. In more complex ecosystems, such as forest, longer sequences (15–20 nt) may be used to improve selectivity [12].

A third key advancement was the identification of susceptibility to unmodified antisense oligonucleotides in insects of the suborder Sternorrhyncha (Hemiptera) [6]. Since then, CUADb-based insecticides have been successfully tested against a wide range of sap-feeding insect pests. These include 28S rRNA targets in *Unaspis euonymi*, *Dynaspidiotus britannicus*, *Icerya purchasi*, *Ceroplastes japonicus*, *Aonidia lauri*, *Coccus hesperidum* [5,12,13,106,107,108,109]; 18S rRNA in *Pseudococcus viburni* [110]; and internal transcribed spacer 2 (ITS2) of pre-rRNA in *Macrosiphoniella sanborni*, *Schizolachnus pineti* [14,111], and *Trioza alacris* [112] (Table 2). ITS2-targeting oligonucleotides have also demonstrated acaricidal potential against the spider mite *Tetranychus urticae* [113,114]. Typically, a single contact treatment with oligonucleotide insecticides at 100 ng/μL results in mortality rates of approximately 80% within 3 to 14 days [12,109]. The first successful application of these insecticides within Sternorrhyncha was conducted on *U. euonymi* in 2019 [107,115].

It is important to note that studies on Sternorrhynchan insects demonstrated that unmodified oligodeoxyribonucleotides (also known as oligonucleotide insecticides or briefly olinscides) can induce both upregulation and downregulation of target genes through a mechanism known as DNA containment (DNAc). This mechanism operates in two sequential steps: the first involves the arrest of target rRNA and/or pre-rRNA (formation of DNA–rRNA duplex), leading to a functional block of ribosomes and triggering a compensatory overexpression of rRNA via rDNA transcription. The second step of DNA containment mechanism involves degradation of the arrested rRNA and/or pre-rRNA by DNA-guided rRNase [5,6,117]. In the presence of rRNase, DNA–rRNA duplex is processed via a mechanism analogous to a zipper, referred to as the ‘genetic zipper’ method [112]. Our recent studies on *C. hesperidum* (4th day after contact application of oligonucleotide insecticide Coccus-11) show that almost all ribosomal proteins of 40S and 60S ribosomal subunits are also significantly upregulated during DNAc promoting the formation of new ribosomes together with hypercompensated rRNA. Ribosome biogenesis proteins involved in the formation of ribosomal subunits (NOP53, UTP30, NSA2, MAK21, BRX1, WDR12) are upregulated as well. Also during DNAc, most of ATP-dependent enzymes are downregulated (including mTOR, serine/threonine protein kinase playing crucial role in ribosome biogenesis through mTORC1 complex) while proteins from mitochondrial ATP synthase complex are upregulated indicating deficiency of cellular energy caused by oligonucleotide insecticide Coccus-11. RNase H1 is also significantly upregulated during DNAc. RNase H1 functions independently of cell cycle and cleaves RNA-DNA hybrids, including those formed between DNA and rRNA. Of note, the innovative ‘genetic zipper’ method not only facilitates accumulation of rRNA and its subsequent degradation as a core process during DNAc but also serves as a predictive algorithm capable of estimating the efficacy of a specific olinscide against the intended target species and closely related organisms possessing identical rRNA target sequences [14,112].

Oligonucleotide insecticides can be designed using DNAInsector program (available at dnainsector.com) or manually, based on pre-rRNA and rRNA sequences retrieved from the GenBank database. In practical terms, this means that individuals with basic sequence knowledge can design an olinscide complementary to the pre-rRNA or rRNA of a Sternorrhynchan insect with a high probability of success. However, to ensure species specificity, it is essential to compare homologous sites in non-target organisms to prevent off-target effects. Synthesis of oligonucleotide insecticides is typically performed using the phosphoramidite method, in either liquid-phase or solid-phase formats. Commonly used solid-phase synthesizers include the ASM-800 (BIOSSET, Novosibirsk, Russia), OligoPilot^TM^ (Cytiva, Uppsala, Sweden), and the 10-Column DNA Synthesizer (PolyGen, Langen, Germany) [108]. The standard preparation involves dissolving the olinscide in nuclease-free water at a concentration of 1 mg per 10 mL and applying it to approximately 1 m^2^ of infested plant foliage.

### 3.3. Perspectives and Limitations of CUADb for Insect Pest Control

DNA-based technologies are currently transforming plant protection by enabling the development of novel control agents with advanced characteristics. DNA, being a natural biopolymer, offers enhanced compatibility with ecological systems, including agroecosystems. Its programmability allows the design of highly specific treatments, including insecticidal and acaricidal agents. Moreover, if resistance arises, adaptive strategies can be implemented by simply redesigning olinscides to the target upstream or downstream regions of the resistance-conferring site in pre-rRNA and rRNA sequences [5]. Oligonucleotide insecticides offer several distinct advantages: they exhibit a low carbon footprint, high specificity to target pests, rapid biodegradability in ecosystems, and minimal risk of target-site resistance. Furthermore, their efficacy can now be predicted across different pest species, particularly in phylogenetically related taxa with conserved target sequences [112]. Given these benefits, it is likely only a matter of time before such DNA-based products become mainstream tools in crop protection. The mechanism of action, known as the DNA containment mechanism, is well understood. The phosphoramidite synthesis method has been optimized, and delivery routes (primarily contact-based, as oral delivery remains less effective) have been established. Although cost remains a barrier, the price of DNA insecticides is expected to decrease with further technological advancements. In fact, CUADb-based insecticides are already becoming cost-competitive with conventional chemical alternatives for some pest species [12].

For instance, CUADb has significantly reduced costs in conifer aphid management by adopting liquid-phase DNA synthesis [13,108]. Sumitomo Chemical Co., Ltd. (Tokyo, Japan), a leading developer of liquid-phase synthesis, reportedly offers 1 kg of unmodified 11-nucleotide oligonucleotides for $25,000 [13]. By contrast, synthesizing the same quantity using standard solid-phase methods, more widely used in research laboratories, can cost up to $1 million. At an application rate of 200 L per hectare with an active ingredient concentration of 0.1 mg/L (0.1 ng/mL), the cost per hectare is approximately $0.50 using liquid-phase synthesis [13]. This level of affordability enables frequent field applications. However, for many Sternorrhynchan pests, effective control requires concentrations around 0.05 g/L, raising the cost to approximately $250 per hectare, even with cost-efficient synthesis. Consequently, achieving an optimal balance between cost and efficacy remains essential for widespread adoption of nucleic acid-based insecticides. Current estimates suggest that CUADb-based approaches could be applicable to 10–15% of all insect pest species.

While oligonucleotide insecticides have demonstrated strong efficacy against hemipteran and moderate efficacy against lepidopteran pests, they have shown much lower activity against coleopterans such as *L. decemlineata* [118]. Incorporating adjuvants (e.g., spreaders, adhesives, penetrants, and UV protectants) into formulations may improve performance, though their environmental safety must be thoroughly assessed. Another critical design consideration is the potential for non-canonical base pairing, such as A:C (C:A) and G:U (T:G) interactions [119,120], which may compromise specificity and increase the risk to non-target organisms. Thus, such interactions must be carefully accounted for during olinscide development [13,15]

Although the CUAD-based ‘genetic zipper’ method presents a promising and cost-effective platform for DNA insecticide development, further optimization of production processes and cost-reduction strategies will be necessary for large-scale implementation. Current research is focused on increasing target specificity by designing olinscides for unique rRNA regions, thereby minimizing off-target effects. Additionally, greater insight into olinscide uptake mechanisms by insect cells is needed to enhance efficacy. In conclusion, the ‘genetic zipper’ holds substantial promise for advancing insect pest control. However, its commercialization will depend on overcoming challenges related to delivery efficiency, production cost, resistance management, and environmental safety. Addressing these limitations will be essential for CUADb-based oligonucleotide insecticides to become a sustainable and widely adopted component of modern pest management.

## 4. CRISPR/Cas

### 4.1. History of Discovery of CRISPR/Cas

While the development of RNA interference (RNAi) technology and the CUADb database followed a relatively linear and well-documented trajectory, the emergence of CRISPR/Cas technology was shaped by the cumulative efforts of numerous research groups. This collaborative history makes it difficult to attribute the discovery to any single individual or team. This section provides an overview of the widely accepted scientific milestones in the development of the CRISPR/Cas system, while acknowledging that many contributors may not be named individually.

In 1987, Ishino and colleagues identified a previously unknown repeat sequence in *Escherichia coli*, although its significance was not fully understood at the time [121]. In 1989, Spanish scientist Mojica, while studying the archaeal microbe *Haloferax mediterranei*, observed similar palindromic repeats of approximately 30 base pairs, separated by unique spacer sequences of roughly 36 base pairs. These did not resemble any known repeat families in prokaryotes at the time [122]. Later Mojica and Jansen introduced the term CRISPR to describe these structures [25]. In 2002, Jansen et al. [123] identified Cas (CRISPR-associated) genes located adjacent to CRISPR loci by suggesting a functional relationship. In 2007, Horvath and colleagues provided the first experimental evidence of CRISPR-Cas system’s role in adaptive immunity. They demonstrated that Streptococcus thermophilus required the Cas7 protein for spacer acquisition and that resistance to phages was mantained by the phage-derived spacer, even in the absence of Cas7, indicating its role in spacer acquisition rather than the immunity process itself [124]. Cas9, previously referred to as Cas5, Csn1, or Csx12, which contains two nuclease motifs, HNH and RuvC, and was shown to be essential for phage resistance, confirming its direct role in DNA cleavage and adaptive immunity [125,126,127]. In 2011, Charpentier identified a small RNA in Streptococcus pyogenes known as trans-activating CRISPR RNA (tracrRNA), which is essential for the processing of CRISPR RNAs (crRNAs) and for guiding the Cas9 complex to cleave target DNA [7]. In 2012, Šikšnys et al. published a seminal study in demontsrating that Cas9 could be programmed with custom-designed spacers to cleave specific DNA targets in vitro [24]. Independently, Charpentier and Jennifer Doudna published in Science, that Cas9 could be guided by synthetic crRNAs and tracrRNAs to cleave dsDNA, with each domains cleaving a complementary DNA strand [25]. These foundational studies established the CRISPR/Cas9 system as a robust genome-editing tool. Although Charpentier, Doudna, and Šikšnys are often credited with demonstrating the system’s genome-editing potential, the technology’s development is the product of decades of collaborative scientific progress.

### 4.2. How CRISPR/Cas Works on Insect Pests

**Figure 4:**
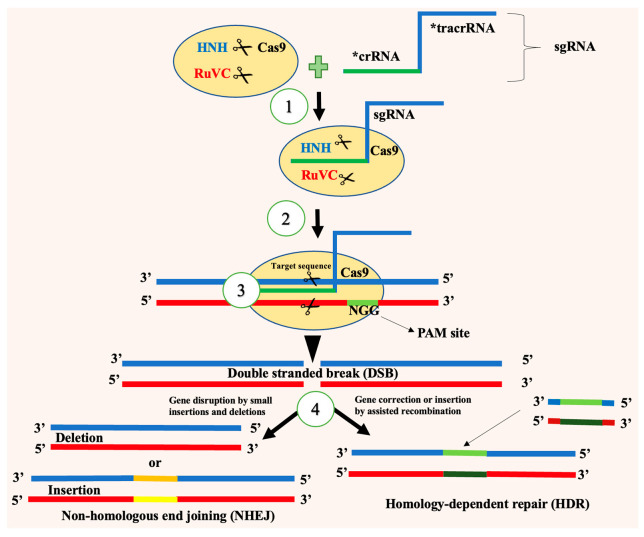
CRISPR/Cas9 system used for insect pest control: 1—sgRNA-Cas9 complex formation; 2—formation of RNA-DNA duplex with target DNA; 3—cleavage of target double-stranded DNA by Cas9; 4—DNA repair through non-homologous end joining (NHEJ) or homologous-dependent repair (HDR); * crRNA—CRISPR RNA; tracrRNA—trans-activating crRNA.

The CRISPR/Cas9 system is the predominant tool in gene editing used for insect pest control, characterized by its simplicity and efficiency. It consists of two key components: a sgRNA (single-guide RNA) and the Cas9 nuclease. The sgRNA is an engineered fusion of CRISPR RNA (crRNA), which defines the target DNA sequence, and trans-activating CRISPR RNA (tracrRNA), which facilitates Cas9 binding. This complex directs Cas9 to specific genomic loci via base pairing. Cas9, a multi-domain DNA endonuclease derived from *Streptococcus pyogenes*, recognizes a protospacer adjacent motif (PAM) sequence—typically 5′-NGG-3′—located near the target DNA site. Upon recognition, Cas9 induces a double-stranded break (DSB) three base pairs upstream of the PAM site. The HNH and RuvC nuclease domains cleave the target and complementary DNA strands, respectively [128,129,130,131,132]. Cellular repair of DSBs occurs via two pathways: non-homologous end joining (NHEJ), which often results in small insertions or deletions that disrupt gene function, or homology-directed repair (HDR), which enables precise gene editing using a repair template) [132,133]. Targeting a gene’s coding sequence with CRISPR/Cas9 and allowing repair via NHEJ often results in a nonfunctional protein. Alternatively, HDR allows for gene knock-ins through integration of exogenous sequences [133,134] (Figure 4).

Since its initial use in *Drosophila*, CRISPR/Cas9 has been widely adopted in diverse insect species. Genome editing aims to introduce engineered traits into wild pest populations, providing a promising strategy to manage invasive pests [135,136]. In the context of insect pests, CRISPR/Cas can be applied in several keyways (Table 3). This approach has been employed to disrupt genes involved in insecticide resistance. A comprehensive review by Xu et al. [137] utlines the application of CRISPR/Cas9 across various insect orders and editing types. Beyond resistance, CRISPR/Cas9 has also been used to manipulate genes regulating reproduction and sex determination. For example, targeting the *Astra-2* (a sex determination gene) in *Anastrepha ludens* results in sterility in males and intersexual phenotypes in females [138]. The *Doublesex (Dsx)* gene, a key regulator of sexual differentiation in insects. has also been edited in species such as diamondback, producing gender-specific sterility through isoform disruption [139]. In addition to these applications, CRISPR/Cas9 has been used to suppress vector competence in disease-transmitting insects. By editing genes essential to pathogen transmission, researchers have successfully reduced the capacity of mosquitoes to spread diseases such as malaria, dengue, and Zika [140,141,142]. Moreover, the system has facilitated the development of gene drives designed to spread deleterious traits through populations, leading to sterility or reduced fitness and, ultimately, population suppression [132,143]. These gene drives bypass Mendelian inheritance through homing endonuclease activity and rely on Cas9 and guide RNAs to propagate engineered alleles during DNA repair [132,144,145,146]. Notably, Meccariello et al. [147] demonstrated the effectiveness of CRISPR/Cas9 gene drives in the Mediterranean fruit fly (*Ceratitis capitata*) by targeting the transformer gene to convert genetic females into fertile XX males. Similar strategies have been explored in beetles, moths and grasshoppers [148,149,150]. In some insect species, such as silkworms, CRISPR-based editing has proven more efficient than RNAi. For instance, knockdown of the homeobox gene *Scr* led to developmental abnormalities and malformed adult structures, showcasing the system’s potential in Lepidoptera [151]. Furthermore, CRISPR/Cas9 has been used synergistically with RNAi technologies. Chloroplast-engineered dsRNA targeting *Frankliniella occidentalis* has been combined with CRISPR/Cas to enhance pest control efficacy [152,153]. In addition to pest management, the technology has significantly advanced functional studies in insects. In *S. frugiperda*, genome editing has expanded our understanding of insect physiology, development, morphology, and behavior [133].

### 4.3. Perspectives and Limitations of CRISPR/Cas for Insect Pest Control

CRISPR/Cas technology presents a promising avenue for insect pest control, offering gene-editing capabilities that may contribute to the development of sustainable pest management strategies, including gene drives and insect-resistant plants. The application of molecular genetic engineering and insect transformation using CRISPR/Cas9 across multiple insect species has helped to overcome numerous limitations associated with traditional approaches, which largely relied on naturally occurring genetic mutations or transposable elements. CRISPR/Cas genome editing is widely favored for its efficiency, simplicity, and adaptability. In addition to Cas9, other Cas proteins, such as Cas3, Cas12a, and Cas13a, have expanded the toolbox for both natural and engineered gene editing, enabling precise modifications, base editing, prime editing, and gene regulation. Recent advancements have also facilitated DNA-free editing, allowing for genome modification without inducing double-stranded breaks [178,179,180].

Despite these developments, several significant challenges remain. It is currently difficult to accurately predict the outcomes of gene editing in insect pests or to determine which gene targets will yield the most effective results. Moreover, CRISPR/Cas systems may introduce unintended edits at off-target genomic sites, which can result in unforeseen biological consequences. Delivering CRISPR/Cas components into insect cells presents another major hurdle, as the efficiency of delivery methods can vary depending on species, developmental stage, or tissue type. Environmental concerns also persist regarding the use of gene drives and insect-resistant organisms, particularly in terms of their potential impacts on non-target species and broader ecosystem dynamics. Gene drives require successful mating between genetically modified and wild-type insects to facilitate the spread of edited traits; however, reproductive barriers, both prezygotic and postzygotic, may restrict gene flow and thus compromise the drive’s efficiency. In addition, genetically modified insects may suffer from reduced fitness, which could limit their ability to compete with wild populations and reduce the long-term success of the strategy. The introduction of precise genetic changes through HDR is also constrained by the technical challenge of delivering sufficient quantities of donor DNA templates [181]. Achieving complex genome modifications remains technically demanding, and issues such as mosaicism, genetic variability among cells in the same organism, may further complicate phenotypic analysis and stable trait inheritance. Although population replacement strategies offer potential benefits, ensuring the long-term sustainability and ecological containment of gene drive systems is essential. Particular attention must be paid to the possibility of unintended spread into non-target species or closely related taxa [133,182]. Furthermore, the long-term effects of CRISPR/Cas-mediated genetic modifications on insect development, behavior, and ecological interactions are not yet fully understood and remain an active area of investigation [137,183].

As research in insect molecular biology continues to progress, CRISPR/Cas technology is poised to become a transformative tool in modern pest management. Its capacity for targeted, species-specific interventions offers a refined and potentially more sustainable alternative to conventional chemical insecticides. Incorporating CRISPR/Cas-based strategies into IPM programs could significantly enhance the precision and resilience of crop protection across diverse agricultural systems, especially when integrated with broader principles of synthetic biology [133].

## 5. Conclusions

Antisense technologies are grounded in natural mechanisms that regulate essential life processes. Complementary interactions between nucleic acids underpin key biological functions such as cell division, metabolism and defense. The development of practical tools based on these antisense mechanisms represents a highly relevant and promising area of scientific research. We believe that sustained, long-term investment in this field is warranted to achieve effective and environmentally friendly solutions for pest management. RNAi, CUADb, and CRISPR/Cas systems are powerful gene silencing tools that operate at distinct molecular levels and offer varying degrees of permanence. RNAi and CUADb transiently reduce gene expression by targeting mRNA and rRNA of pests, respectively, whereas CRISPR/Cas induces permanent gene silencing through targeting alterations of genomic DNA. The CUADb system is unique among these technologies due to its DNA-guided mechanism and involvement in rRNA biogenesis, although it shares certain functional similarities with RNAi and CRISPR/Cas. Collectively, these systems hold considerable promise in overcoming insecticide resistance by targeting conserved genes and supporting the development of effective pest control agents.

The application of these technologies in insect pest control has already demonstrated significant potential. Notably, RNAi, CUADb and CRISPR/Cas exhibit optimal efficacy against specific insect pest groups, highlighting the potential for combining these approaches to enhance their effectiveness across broader pest populations. Although there is some level of competition among these modern antisense technologies, the physiological and genetic diversity of insect pests indicates that no single approach will provide a universal solution. Currently, antisense-based insect pest control technologies remain in the development phase. As data on their successes and limitations continue to accumulate, major companies are actively pursuing commercialization. Meanwhile, legislative framework governing the implementation of antisense technologies are gradually evolving in various countries. Although rapid breakthroughs may not be imminent, steady progress is inevitable, and will ultimately contribute to improvements in both human health and environmental sustainability.

In conclusion, RNAi, CUADb, and CRISPR/Cas represent three complementary ‘worlds’ of antisense technologies with promising future applications in diverse fields. These technologies hold potential for improving agriculture and developing new pest control strategies. CRISPR/Cas can create mutations for long-term changes, while RNAi and CUADb provide temporary gene silencing to block certain gene function or quickly adapt to resistance of insect pests. This combination of antisense tools offers versatility in addressing various aspects of pest control, including resistance management, population suppression, and developing novel and eco-friendly pest management strategies. However, challenges related to delivery, affordability, and environmental considerations need to be addressed for these technologies to reach their full potential.

## Figures and Tables

**Table 1:** List of pest species successfully targeted by RNAi.

Sl. No.	Names of Model Insects	Targeted Gene(s)	Affected Processes	References
1	Beet armyworm*, Spodoptera exigua* (Lepidoptera)	*Chitin synthase gene A*	Chitin synthesis	[40]
2	Brown planthopper, *Nilaparvata lugens* (Hemiptera)	*NlHT1*, *Nlcar*, *Nltry* *NlTPS*	Digestive system Enzymatic activity	[41,42]
3	African sweet potato weevil, *Cylas puncticollis* (Coleoptera)	*Snf7*	Digestive system	[43]
4	Tomato pinworm, *Tuta absoluta* (Lepidoptera)	*Vacuolar ATPase-A and Arginine kinase*	High mortality	[44]
5	Oriental fruit fly, *Bactrocera dorsalis* (Diptera)	*α-Spectrin*	Oviposition and ovary size	[45]
6	Cotton mealybug, *Phenacoccus solenopsis* (Hemiptera)	*Krüppel homologue-1*, *ADP-ATP/Translocase*, *IDGF-1* *Bursicon, V-ATPase*	Not specified Cuticle hardening and V-ATPases act as proton pumps	[46,47]
7	Diamondback moth, *Plutella xylostella* (Lepidoptera)	*PxCht*	Chitin synthesis	[48]
8	Fall armyworm, *Spodoptera frugiperda* (Lepidoptera)	*Met*, *EcR*, *USP* *COPIα*, *COPIβ*, *GSTU1*	Reproductive system, fertility Insect reproduction	[49,50]
9	White-backed planthopper, *Sogatella furcifera* (Hemiptera)	*hsc70-3*, *PP-α*	Insect metamorphosis	[51]
10	Soybean aphid, *Aphis glycines* (Hemiptera)	*Cytochrome P450 monooxygenases (CYP450s)*	Insect resistance	[52]
11	Asian citrus psyllid, *Diaphorina citri* (Hemiptera)	*CHC*, *vATPase-A*, *Snf7*	Transmembrane system	[53]
12	*Trichogramma dendrolimi* (Lepidoptera)	*Vitellogenin receptor (VgR)*	Female reproductive system	[54]
13	Domestic silk moth, *Bombyx mori* (Lepidoptera)	*BmToll9-1*, *BmToll9-2*, *PGRP-L4*	Toll and immune deficiency signaling pathways	[55,56,57,58]
14	Silverleaf whitefly*, Bemisia tabaci* (Hemiptera)	*Cysteine protease*	Digestive system	[59]
15	Cowpea weevil, *Callosobruchus maculatus* (Coleoptera)	*Olfactory receptor coreceptor (Cmac\Orco)*	Insect sensory system	[60]
16	White-backed planthopper, *S. furcifera* (Hemiptera)	*β-N-acetylhexosaminidase genes*	Insect metamorphosis	[61]
17	Desert locust, *Schistocerca gregaria* (Orthoptera)	*Cytochrome P450*	Ecdysteroid pathway	[62]
18	Red flour beetle, *Tribolium castaneum* (Coleoptera)	*CPAPs*	Cuticular proteins	[63]
19	Chinese white pine beetle, *Dendroctonus armandi* (Coleoptera)	*Aquaporin*	Osmoregulation	[64]
20	Kissing bug, *Rhodnius prolixus* (Hemiptera)	*Nitrophorin 2 (NP2)*	Anticoagulant and apyrase activities in saliva	[65]
21	Citrus aphid, *Toxoptera citricida* (Hemiptera)	*TCiCHS*	Chitin synthesis	[66]
22	Potato psyllid*, Bactericera cockerelli* (Hemiptera)	*SUC1*, *ST4*	Osmoregulatory	[67]

**Table 2:** List of pest species successfully targeted by CUADb.

Sl. No.	Names of Model Insects	Targeted Gene(s)	Affected Processes	References
1	Euonymous scale*, U. euonymi* (Hemiptera)	*28S rRNA*	Protein biosynthesis	[107,115]
2	Holly scale, *D. britannicus* (Hemiptera)	*28S rRNA*	Protein biosynthesis	[5,107]
3	Japanese wax scale, *C. japonicus* (Hemiptera)	*28S rRNA*	Protein biosynthesis	[106]
4	Cactus scale, *Diaspis echinocacti* (Hemiptera)	*28S rRNA*	Protein biosynthesis	[116]
5	Bay sucker, *T. alacris* (Hemiptera)	*ITS2 of pre-rRNA and 28S rRNA*	Protein biosynthesis	[112]
6	Cottony cushion scale*, I. purchasi* (Hemiptera)	*28S rRNA*	Protein biosynthesis	[108]
7	Chrysanthemum aphid*, M. sanborni* (Hemiptera)	*ITS2 of pre-rRNA*	Protein biosynthesis	[111]
8	Mealybug*, P. viburni* (Hemiptera)	*5.8S, 18S and 28S rRNA*	Protein biosynthesis	[110]
9	Laureal scale, *A. lauri* (Hemiptera)	*28S rRNA*	Protein biosynthesis	[5]
10	Soft scale*, C. hesperidum* (Hemiptera)	*28S rRNA*	Protein biosynthesis	[105]
11	Two-spotted spider mite, *T. urticae* (Trombidiformes)	*ITS2 of pre-rRNA*	Protein biosynthesis	[113]
12	Grey pine aphid*, S. pineti* (Hemiptera)	*ITS2 of pre-rRNA*	Protein biosynthesis	[13]
13	Large pine aphid, *Cinara pinea* (Hemiptera)	*ITS2 of pre-rRNA*	Protein biosynthesis	[13]
14	Pine needle aphid, *Eulachnus rileyi* (Hemiptera)	*ITS2 of pre-rRNA*	Protein biosynthesis	[13]

**Table 3:** List of insect species successfully targeted by CRISPR/Cas system.

Sl. No.	Names of Model Insects	Target Gene(s)	Affected Processes	Reference
1	Mosquito, *Anopheles stephensi* (Diptera)	*Kynurenine hydroxylase*	Parasite-resistance	[154]
2	Fall armyworm, *S. frugiperda* (Lepidoptera)	*Ebony* *Doublesex (dsx) (Sfdsx)* *Antennapedia (Antp)* *Spermatogenesis-related, tssk2*	Melanin biosynthesis Sex differentiation Insect thorax and wing development Male reproductive system	[155,156,157,158]
3	Diamondback moth, *P. xylostella* (Lepidoptera)	*Yellow* *Ebony* *LW-opsin*	Body pigmentation Body pigmentation Efficiency of phototaxis	[46,159,160]
4	European bee, *Apis mellifera* (Hymenoptera)	*Amyellow-y*	Melanization in cuticle	[161]
5	Beet armyworm, *S. exigua* (Lepidoptera)	*Desaturase (SexiDES5)*	Sex pheromone biosynthesis	[162]
6	Brown planthopper, *N. lugens* (Hemiptera*)*	*Cysteine sulfinic acid decarboxylase (CSAD)*	Melanin metabolism	[163]
7	Chickpea pod borer, *H. armigera* (Lepidoptera)	*Wnt1*	Segmentation, appendage development, and pigmentation	[164]
8	Asian corn borer, *Ostrinia furnacalis* (Lepidoptera)	*Abdominal-A (Abd-A) and Ultrabithorax (Ubx)*	Anatomical structure formation	[165]
9	Black garden ant, *Lasius niger* (Hymenoptera)	*Cinnabar*	Eye pigmentation	[166]
10	Common cutworm, *S. litura* (Lepidoptera)	*Serine protease 2* *Odorant-binding proteins*	Male sterility Perception of a sex pheromone	[153,167]
11	Indian meal moth, *Plodia interpunctella* (Lepidoptera)	*ATP binding cassette (ABC) proteins*	Eye pigmentation	[168]
12	Eggplant shoot and fruit borer, *Leucinodes orbonalis* (Lepidoptera)	*Tryptophan 2, 3-dioxygenase* *Vitellogenin (Vg)*	Eye pigmentation Female reproductive system	[169,170]
13	Mango fruit fly, *B. dorsalis* (Diptera)	*White* *White locus* *OBP13*	Eye pigmentation Eye pigmentation Methyl eugenol	[171,172,173,174]
14	Pomace fly, *Drosophila suzukii* (Diptera)	*Doublesex*	Population suppression	[175]
15	Australian cotton bollworm, *H. armigera conferta* (Lepidoptera)	*Cadherin*	Cry1Ac resistance	[176]
16	Cricket, *Gryllus bimaculatus* (Orthoptera)	*Laccase 2 (Gb-lac2)*	Cuticle system pigmentation	[177]

## Data Availability

No new data were created.
